# The generation and evaluation of TKO/hCD55/hTM/hEPCR gene-modified pigs for clinical organ xenotransplantation

**DOI:** 10.3389/fimmu.2024.1488552

**Published:** 2025-01-20

**Authors:** Guoli Huai, Yong Wang, Jiaxiang Du, Zhenhui Cheng, Yuxuan Xie, Jia Zhou, Hongmei Tang, Yanyan Jiang, Xiangyang Xing, Shaoping Deng, Dengke Pan

**Affiliations:** ^1^ Clinical Immunology Translational Medicine Key Laboratory of Sichuan Province, Sichuan Provincial People's Hospital, School of Medicine, University of Electronic Science and Technology of China, Chengdu, China; ^2^ Chengdu ClonOrgan Biotechnology, Co., Ltd., Chengdu, China; ^3^ Sichuan ClonOrgan Biotechnology, Co., Ltd., Neijing, China

**Keywords:** genetically modified pigs, xenotransplantation, TKO/hCD55/hTM/hEPCR, infection, DPF facility

## Abstract

**Introduction:**

Genetically edited pigs, modified using CRISPR-Cas9 technology, hold promise as potential sources for xenotransplantation. However, the optimal combination of genetic modifications and their expression levels for initial clinical trials remains unclear. This study investigates the generation of TKO/hCD55/hTM/hEPCR (6GE) pigs and evaluates their compatibility with human immune and coagulation systems.

**Methods:**

The 6GE pigs were generated through iterative genome editing and F1 generation breeding. Genotyping, flow cytometry, and immunohistochemistry confirmed the knockout of GGTA1, CMAH, and B4GALNT2. Expression levels of human genes (hCD55, hTM, hEPCR) were quantified. In vitro assays using aortic endothelial cells (pAECs) from 6GE pigs assessed human serum IgM and IgG binding, complement cytotoxicity, and thrombin-antithrombin (TAT) complex levels. Blood from gene-edited pigs was used for pathophysiological analysis.

**Results:**

Complete knockout of GGTA1, CMAH, and B4GALNT2 was confirmed in 6GE pigs. The expression of hCD55 and hTM was approximately seven and thirteen times higher than in humans, respectively, while hEPCR levels were comparable to those in humans. *In vitro*, 6GE pAECs showed significantly reduced binding of human IgM and IgG compared to wild-type pAECs (IgG p<0.01, IgM p<0.0001). Similar to TKO/hCD55 pAECs, 6GE pAECs exhibited a substantial reduction in complement-mediated cytotoxicity (p<0.001) compared to TKO pAECs. Co-expression of hTM and hEPCR in 6GE pigs led to a significant decrease in thrombin-antithrombin (TAT) complex levels in co-culture with human whole blood, compared to WT (p<0.0001), TKO (p<0.01), and TKO/hCD55/hTM pigs (p<0.05). Pathophysiological analysis demonstrated excellent compatibility of 6GE pig kidneys and livers with human immune and coagulation systems. However, 6GE pigs showed increased susceptibility to infection compared to other gene-edited pigs, while TKO/hCD55 pigs were considered safe when they were all bred in a general environment.

**Discussion:**

Highly expressing hCD55, along with the co-expression of hEPCR and hTM genes, is expected to effectively reduce human complement cytotoxicity and enhance anticoagulant efficacy in genetically modified pigs. The 6GE pigs exhibited robust compatibility with human physiological and immune systems, fulfilling the criteria for clinical trials. Furthermore, it is imperative to rear donor pigs in pathogen-free (DPF) facilities to mitigate infection risks and prevent the transmission of porcine pathogens to humans.

## 1 Introduction

Organ transplantation stands as the only effective treatment for end-stage organ failure. However, the shortage of donor organs severely impedes its clinical application and advancement (1). Xenogeneic tissues or organs represent a significant avenue to address this challenge. Due to similarities in organ size, anatomy, and physiology with humans, as well as ease of breeding, pigs are considered the primary candidates as xenogeneic organ sources (2). Nonetheless, the interspecies incompatibility between pig-derived grafts and humans in immunological and physiological aspects leads to hyperacute rejection (HAR), posing a critical obstacle to the survival of xenografts (3). With the rapid development of CRISPR/Cas9 technology, a multi-gene editing strategy can be employed to modify donor pigs to overcome the HAR seen after xenotransplantation (4, 5).

The factors causing HAR is that porcine xenoantigens, e.g., galactose-alpha-1,3-galactose (α-Gal) are recognized and bound by natural (preformed) antibodies in the human serum (6). Recent advances in genome editing have paved the way for the creation of pigs with gene disruptions causing multiple xenoantigen knockouts (KO). CRISPR/Cas9 was used to create triple gene KO (TKO) pigs (α-Gal, Neu5Gc and SDa KO). TKO porcine cells exhibit markedly reduced binding of human natural antibodies (7). In preclinical pig-to-primate studies, simultaneous inactivation of the GGTA1 and CMAH genes increases non-human primate antibody binding (8). Anti-TKO IgM was significantly higher in Old World monkeys (OWMs) than in humans and cytotoxicity of OWM sera to TKO PBMCs was significantly greater than of human serum (9, 10). To summarize, TKO pigs are ideal donors for humans but not for OWMs.

In addition to the expression of the major xenoantigens in pigs, antibody-mediated rejection (AMR) was also triggered by activation of the complement cascade, resulting the xenograft failure (11, 12). Therefore, the additional expression of several human transgenic proteins to block rejection induced by completement would be beneficial (13). The human complement regulatory proteins (hCRPs) include CD46 (membrane cofactor protein), CD55 (decay-accelerating factor) and CD59 (MAC-inhibitory protein). Expression of one or more hCRPs has been shown to prolong the graft survival (14, 15).

Furthermore, thrombotic microangiopathy and systemic consumptive coagulopathy were also observed in xenotransplantation (16). Thus, editing the genes related to coagulation dysregulation in the donor pig is essential to promote successful xenotransplantation (17). Thrombomodulin (TM) is a crucial cofactor to activate protein C and inhibit the coagulation cascade. Grafts from hTM transgenic pigs are more effective in activating protein C than those from wild-type pigs (18, 19). Endothelial protein C receptor (EPCR), as a cofactor to enhance the activation of protein C, is also an important gene to ameliorate coagulation problems (20). pAECs expressing hEPCR induced less platelet aggregation. The combination of transgenic expression of EPCR and TM in pigs may reduce the anticipated coagulation dysregulation (21).

Currently, a total of 14 cases of xenotransplantation from gene-edited pigs to humans (some of them in decedents) have been reported, including 9 kidney transplants, 1 liver transplant, and 4 heart transplants (22–25). Among these cases, the latest kidney xenotransplant involved a gene-modified pig with 69 edited genes, resulting in the successful discharge from hospital of the patient (26). The gene modifications in the above donor pigs consisted of triple knockout of xenoantigens, and insertion of human complement-regulatory proteins (hCRPs) and anticoagulant genes. However, it is crucial to note that more genetic editing does not necessarily equate to better outcome. The selection of gene combinations and the expression levels of genes significantly affect the final results. What’s more, the multiple transgenic pigs may be beneficial in preventing primate immune response, they are also more susceptible to infection. The purpose of genetic editing is to enhance compatibility with the human recipient and promote long-term survival of the grafts. Therefore, through this present paper, we introduce our TKO/hCD55/hTM/hEPCR (6GE) gene-edited pigs for functional assessment and suggest they are ideal donors for clinical transplantation and emphasize the necessity of a DPF facility for clinical applications. This is essential not only to mitigate their susceptibility to infection but also to prevent the transfer of pig pathogens to humans (zoonosis).

## 2 Materials and methods

### 2.1 Pigs welfare

Animal studies were conducted with the approval of the Institutional Animal Care and Use Committee (IACUC) of Sichuan Provincial People’s hospital (AF/22.01.0). Chinese Bama minipigs (25~40 kg) and mature Landrace pigs (average body weight ~105 kg) were kindly provided by and housed in a large-scale pig facility operated by Chengdu Clonorgan Biotechnology Co., Ltd. All animals were managed under standard husbandry practices, and daily health monitoring to ensure their well-being. Following the collection of blood samples, ear tissue, and other specimens, gene-edited Bama minipigs were humanely euthanized via intravenous injection of an overdose of pentobarbital, ensuring rapid and stress-free death. Organs and tissues, such as kidneys and aortas, were subsequently harvested. Upon completion of all experiments, animal carcasses were incinerated at a licensed facility in compliance with biosafety and environmental protection regulations.

### 2.2 Human and pig blood preparation and analyses

Human Blood samples were obtained from 20 healthy volunteers (aged 22–44 years, both male and female) with no documented history of prior exposure to porcine antigens or alloantigens. A fraction of each sample was collected in anticoagulant-free tubes and immediately used for experimental procedures. Another fraction was processed to separate serum, which was used for biochemical assays, with the remaining serum aliquoted and stored at -80°C for future analyses. Additionally, blood was collected in EDTA tubes for routine hematological evaluations. In the same way, wild-type (WT) and genetically modified pigs blood samples were harvested for biochemical assays and routine hematological evaluations.

### 2.3 Generation of guide RNA for gene targeting

The genomic sequences of target genes GGTA1, B4GALNT2/B4GALNT2 LIKE, and CMAH were retrieved from the NCBI database. Guided RNA (gRNA) designs were performed using the CRISPRCAS9 design website (https://zlab.bio/guide-design-resources). Upon completion of the design process, gRNA-related primers were synthesized by BGI Genomics Co., Ltd. Specifically, the GGTA1 gene (NCBI Gene ID: 396733) was targeted at exon 4 with the sequence 5’-GAGAAAATAATGAATGTCAA-3’, while CMAH (NCBI Gene ID: 396918) was targeted at exon 1 with the sequence 5’-GTTCTTACATGCCTTCAGG-3’. For B4GalNT2 (NCBI Gene ID: 100621328) and B4GalNT2-like (NCBI Gene ID: 110255214), genes sharing similar sequences, a single gRNA was identified capable of knocking out exon 2 for both genes, with the sequence 5’-GGGACGGGATGGGTGAGTTG-3’. Subsequently, the three gRNAs were ligated into the knockout plasmid pX458 (Solarbio, VT000062). Following sequence verification to confirm correct bacterial strains, plasmids were cultured and extracted for further experimentation.

### 2.4 Porcine primary ear fibroblasts isolation and culture

We harvested 1-2 cm³ of pig ear tissue, soaked the tissue in alcohol for 2-5 minutes, then transferred it to DMEM without FBS. In a 60 mm dish, the tissue was opened to expose cartilage, fat, etc., which was scraped off, leaving only the epidermis. Using scissors to cut the epidermis into small pieces, we added 2 mL of FBS to resuspend them, and evenly distributed the tissue fragments onto the slanted surface of a T-25 flask. Along the other side, we added 5 mL of DMEM containing 20% FBS and 5% penicillin-streptomycin. The flask was placed a cell culture incubator with the tissue fragments facing upwards. After approximately 8 hours, the flask was inverted gently to allow the culture medium to soak the tissue. The culture medium was changed every 2 days. After around 7 days, cells started migrating out. The tissue fragments were removed and the cells transferred to a 10 cm culture dish for further culture.

### 2.5 Construction of gene editing vector

mRNA was extracted from a human aortic endothelial cell line. The TransScript^®^ IV One-Step gDNA Removal and cDNA Synthesis SuperMix (Transgen biotech, AW311-02) was used to obtain cDNA. The sequences of hCD55 (NM_000574.5), hTM (NM_000361.3), and hEPCR (NM_006404.5) were amplified, and subsequently connected to a promoter and bGH polyA sequence. hCD55 was integrated into the pig Rosa26 locus to construct a pig site-specific integration targeted deletion plasmid. The gRNA sequence for the Rosa26 locus was 5’- GCCCAAGGAGACCTGGAGAA-3’, and for the COL1A1 locus was 5’- GCCCAAGGAGACCTGGAGAA-3’. Pig genomic DNA was extracted from pig ear fibroblasts using a genome DNA extraction kit (TIANMO biotech, #TD468). Upstream and downstream sequences of 1000 bp each from the deletion target were amplified as homologous arms. After correct sequencing, these sequences were connected to the left and right ends of the vector, with HSV-TK sequence serving as a negative selection marker, used to eliminate randomly integrated cell clones.

### 2.6 Transfection and selection of positive cell colonies

Two days prior to transfection, primary pig ear fibroblasts were seeded into 60 mm dishes and cultured until reaching 70-80% confluency. Transfection was performed using an electroporation kit (Lonza, VPI-1002). After electroporation, cells were cultured in 60 mm dishes until reaching 80% confluency. The original culture medium was discarded, and the cells were washed 1-2 times with DPBS according to standard procedures. After washing, an appropriate amount of 0.1% trypsin was added, and the dishes were placed in a cell culture incubator for 2 minutes. Trypsin digestion was terminated by adding an appropriate amount of DMEM media at 3-5 times the volume of trypsin. Then, 1 mL of DMEM media was added to resuspend the cells, and cell counting was performed. Based on the cell count, the cell suspension was diluted and seeded into 5 to 10 cell culture dishes (100mm) at a certain density (1000-2000 cells per dish). Each dish was supplemented with 10 mL of DMEM medium and placed in an incubator. DMEM medium was replaced every 24 hours, supplemented with ganciclovir (MedChemExpress, #HY-13637). The medium was changed every 2 days. After 10-12 days, single cells were obtained. Single-cell clones were seeded into a 96-well plate, and after isolation of cell DNA, PCR identification was performed. PCR amplification and sequencing were conducted using primers targeting the vicinity of the deletion genes GGTA1, B4GALNT2/B4GALNT2 LIKE, and CMAH (**Table 1**). Integration of the genes was identified by PCR amplification using primers for the left and right homologous arms. L-F/R and R-F/R primers were used to identify integration at the left and right ends of the integration site, respectively. GAPDH was used as an internal reference for DNA.

**Table 1:** The list of gene sequence and length of the primers.

Primer Name	Sequence (5’ to 3’)	Sequence length(bp)
GGTA1-F	AGGGACAGTAGACCTAGGAAAC	652
GGTA1-R	GATCCTAATTGGGTTTGCTGCC	
B4GalNT2-F	GCTATTCCCATCTATGTCGCA	283
B4GalNT2-R	TCTCACCCGTTTTCACCGT	
CMAH-F	GACCTGTGGAGCTGTCAATGCTC	724
CMAH-R	GCTCTGCCATTTTTCGGGTTTTCA	
hCD55-Rosa26-L-F	GCATATCGTTTGTTACGCTGG	1419
hCD55-Rosa26-L-R	ACGGCACTTACCTGTGTTCTGG	
hCD55-Rosa26-R-F	CTAGAGCTTGGCGTAATCATGGTCA	1747
hCD55-Rosa26-R-F	GTCACAAAAGCCATACTTCCAAGG	
hTMEPCR-COL1A1-L-F	ACCGGGTTCGGAGGAAAGTC	1343
hTMEPCR-COL1A1-L-R	CACGGACCAGCTTTCCTGAACT	
hTMEPCR-COL1A1-R-F	CCTCCAAAGACTTCATATGCTCCA	2179
hTMEPCR-COL1A1-R-R	ATCTGTGCCGGCTCTTGTT	
GAPDH-F	TCTGGCAAAGTGGACATTGT	973
GAPDH-R	CCCTCCTCTGATGTCCTGA	

### 2.7 Somatic cell nuclear transfer and embryo transfer for pig cloning

The positive cloned cells were selected and seeded into 60 mm culture dishes. When they reached 80% confluence, the cells were digested with trypsin and resuspended for later use. Ovaries were collected and ovaries harvested, then cultured for 30-40 hours. 0.1% hyaluronidase solution (SIGMA, #37326-33) was used to remove cumulus cells. Mature oocytes were selected with intact zona pellucida, clear perivitelline space, and extruded first polar body. The positive cloned cells were resuspended in 0.1% hyaluronidase solution for later use. The denuded oocytes were transferred to a microdrop and the first polar body was aspirated along with 10-20% of cytoplasm, which may contain oocyte nuclei. Round, smooth, and strong refractive body cells with a diameter of 15-20 µm were selected. The donor cell was placed into the perivitelline space through the enucleation incision and pressed gently with an injection needle to ensure close contact between the donor cell and the oocyte membrane. The reconstructed oocytes (reconstructed embryos) were transferred to an embryo culture medium containing 4 mg/ml BSA and cultured in an incubator at 38.5°C with 5% CO2 and 100% humidity for 1.5 hours. Subsequently, somatic cell nuclear transfer was carried out. The reconstructed embryos cultured from the previous day were inspected under a stereomicroscope. Poorly developed and dead reconstructed embryos were discarded. The healthy ones were transferred to cryopreservation tubes containing culture media. After sealing the cryopreservation tubes, they were placed in an embryo transfer box preheated to 38.5°C. We confirmed that the recipient sow’s ovaries met the transplantation requirements. The reconstructed embryos were surgically transferred into the pig oviducts.

### 2.8 Genotyping of cloned pig

A small piece of ear tissue was excised from a 7-days-old cloned piglet. The tissue was ground using a tissue grinder. Pig genomic DNA was extracted using a genomic DNA extraction kit (TIANMO biotech, TD468). PCR was performed for DNA identification using the same method and primers as used for screening positive cloned cells.

### 2.9 Isolation and culture of porcine aorta endothelial cells

Pig aorta was harvested under aseptic condition. Porcine aortic endothelial cells (pAEC) were isolated and cultured based on the Yanli Zhao’s method (27). In brief, we gently cut off the excess tissues and arterial side branches around the aorta with sterile forceps and scissors. We washed the outside and inside of the aorta with DPBS. A surgical suture (5-0, 90cm) was placed in the inside of the aorta, and then the surgical suture was pulled slowly to reverse the aorta to expose the endothelial surface of the aorta. After washing, the aorta was digested by 1mg/ml of 0.005% collagenase I (Gibco, 17018029). After incubation at 37°C for 15 min, the digestion was stopped by stopping buffer. We harvested the digestion solution and gently scraped the surface of the aorta for more pAECs, which were washed. The digested liquid was collected, centrifuged, and the cell pellets were resuspended with 1 ml ECM medium (ScienCell, #1001) with 10% FBS, 1% penicillin/streptomycin (P/S) and 1% endothelial cell growth supplement (ECGS). The cells were cultured and the medium replaced every 2−3 days. When the pAECs filled the culture container, 0.5% trypsin solution (ThermoFisher, 15050065) was added for digestion. The pAECs’ pellet was resuspended with DPBS solution, and CD31 antibody (Mouse anti-pig FITC-CD31 (Biolegend, MCA1746F, 1:100) was added to stain and test the purity.

### 2.10 Analysis of transgenic expression at the protein level using FACS

pAECs were isolated from 6GE and WT pigs, HUVECs were used as the control. The gene modifications of the cells were analyzed using FACS at the protein level. According to manufacturer’s instructions, cells were collected and stained with primary and secondary antibodies. aGal was stained by FITC conjugated Isolectin B4 (BSI-B4, SIGMA, L2895). B4GalNT2 phenotype was carried out using Fluorescein Dolichos Biflorus Agglutinin (DBA, Vector Laboratories, FL-1031). The expression level of Neu5Gc was assessed by chicken anti-Neu5Gc antibody (BioLegend, #146901), the secondary antibody was Alexa Fluor488 goat anti-chicken (Abcam, ab96947), and the chicken IgY Isotype was used as negative control (BioLegend, 402101). hCD55, hTM and hEPCR were determined by mouse anti-human PE-CD55 (santa cruz, SC-59092, 1:100), mouse anti-human APC-TM (BD, 564123, 1:200), mouse anti-human AF488-EPCR (BD, 563623, 1:200). Samples were washed and analyzed on a flow cytometer (CytoFLEX, Beckman). The data were collected and analyzed using FlowJo software (Flowjo_v10.8).

### 2.11 Characterization of protein expression by immunofluorescence and immunohistochemistry

The kidneys were harvested from 6-GE and WT pigs and snap-frozen in liquid nitrogen. Then the frozen kidney was embedded in optimal cutting temperature (OCT) compound and cryo-sectioned into 4 μm thickness. All the sections were air dried, acetone fixed and incubated with antibodies. TKO was verified by immunofluorescence. To detect the aGal epitope, the sections were stained with BSI-B4-Alexa 488 (SIGMA, L2895,1:500). For the detection of SDa, DBA (Vector Laboratories, FL-1031) was used. For the detection of Neu5Gc, the sections were incubated overnight with the primary antibody (chicken anti-Neu5Gc polyclonal antibody (BioLegend, 146901, 1:1500) at 4°C and were subsequently incubated for 2 h at room temperature with an Alexa Fluor 488-conjugated goat anti-chicken secondary antibody (Abcam, ab96947, 1:1000). Nuclei were counterstained with 5mg/ml 6-diamidino-2-phenylindole (DAPI, Biotechnology, 1:1000). The slides were examined using fluorescence microscopy (Nikon, Tokyo, Japan). CD55/TM/EPCR expression was tested by immunohistochemistry. CD55 was stained by anti-CD55 antibody (Abcam, ab133684). TM was tested used TM antibody (Santa Cruz Biotechnology, sc-13164). EPCR was determined by anti-EPCR/CD201 antibody (Abcam, ab300565). After primary antibody staining, the corresponding secondary antibodies were used. Nuclei were counterstained with a fluorescence microscope (Nikon, TS2-FL).

### 2.12 Antibody binding of human and porcine endothelial cells

IgM and IgG binding assays were carried out on pAECs using mixed human serum of all ABO blood types. Human sera were obtained from volunteers with IRB approval. WT, TKO(3GE), TKO/CD55(4GE), TKO/CD55/hTM(5GE), TKO/CD55/hTM/hEPCR(6GE) pAECs and HUVECs (1×10^5^ cells/each) were harvested and resuspended in PBS (1% BSA), adding the final 25% of heat-inactivated human serum as the sample control group, and adding PBS for the antibody control group. The cells were mixed with medium and incubated for 30 min at 4°C. Cells were washed and blocked with 10% goat serum (Sigma-Aldrich) for 30 min at 4°C. After that, cells were stained with Alexa fluor488-conjugated Affipure goat anti-human IgG(H+L) (Jackson immunoresearch, 109-545-003, 1:1000), Alexa fluor647-conjugated goat anti-human IgM (Jackson immunoresearch, 109-605-043, 1:1000) for 30 min at 4°C to detect IgM or IgG binding. Sample analysis was completed on a flow cytometer and data were analyzed using FlowJo analysis software. The antibody binding results were reported as median fluorescence intensity (MFI).

### 2.13 Complement-dependent cytotoxicity

WT, 3GE, 4GE, 5GE, 6GE pAECs and HUVECs were collected and resuspended in serum-free culture medium. Cells (1×10^5^ cells/each) were mixed with 25% human serum as the sample control group, adding serum-free culture medium as the control group, and then incubated for 1h at 37°C. Cells were then stained with propidium iodide (PI, Invitrogen, P3566) for 5min and analyzed using a flow cytometer. The cell death rate was calculated as: relative cytotoxicity = the rate of PI-positive cells of sample group - the rate of PI-positive cells of control.

### 2.14 TAT assay for coagulation system test

pAECs of WT, 3GE, 4GE, 5GE, 6GE and HUVECs were harvested and seeded at 5×10^4^/well in 48-well plates. When all the cells covered 90% of the bottom of the well, the medium was dropped and washed, and then 200μL of fresh whole human blood were added and incubated at 37°C with gentle shaking for 15min, 30min, 45min, and 1h. At the different time-points, all the blood was collected, plasma was isolated and preserved at -80°C. After repeating three times, all the samples were measured using a Human Thrombin–Antithrombin Complex ELISA Kit (Ruixinbio, RX105411H).

### 2.15 Statistical analysis

Graph and data were analyzed with Microsoft Excel and completed using Prism 8 for windows (GraphPad Software Inc. La Jolla, CA, USA). P values were determined by using student’s t tests and ANOVA for all quantifications. P<0.05 was typically used as statistically significant.

## 3 Results

### 3.1 Generation of pigs with gene‐edited knockout of GGTA1/CMAH/B4GALNT2 and transgenes of hTM/hCD55/hEPCR

**Figure 1:**
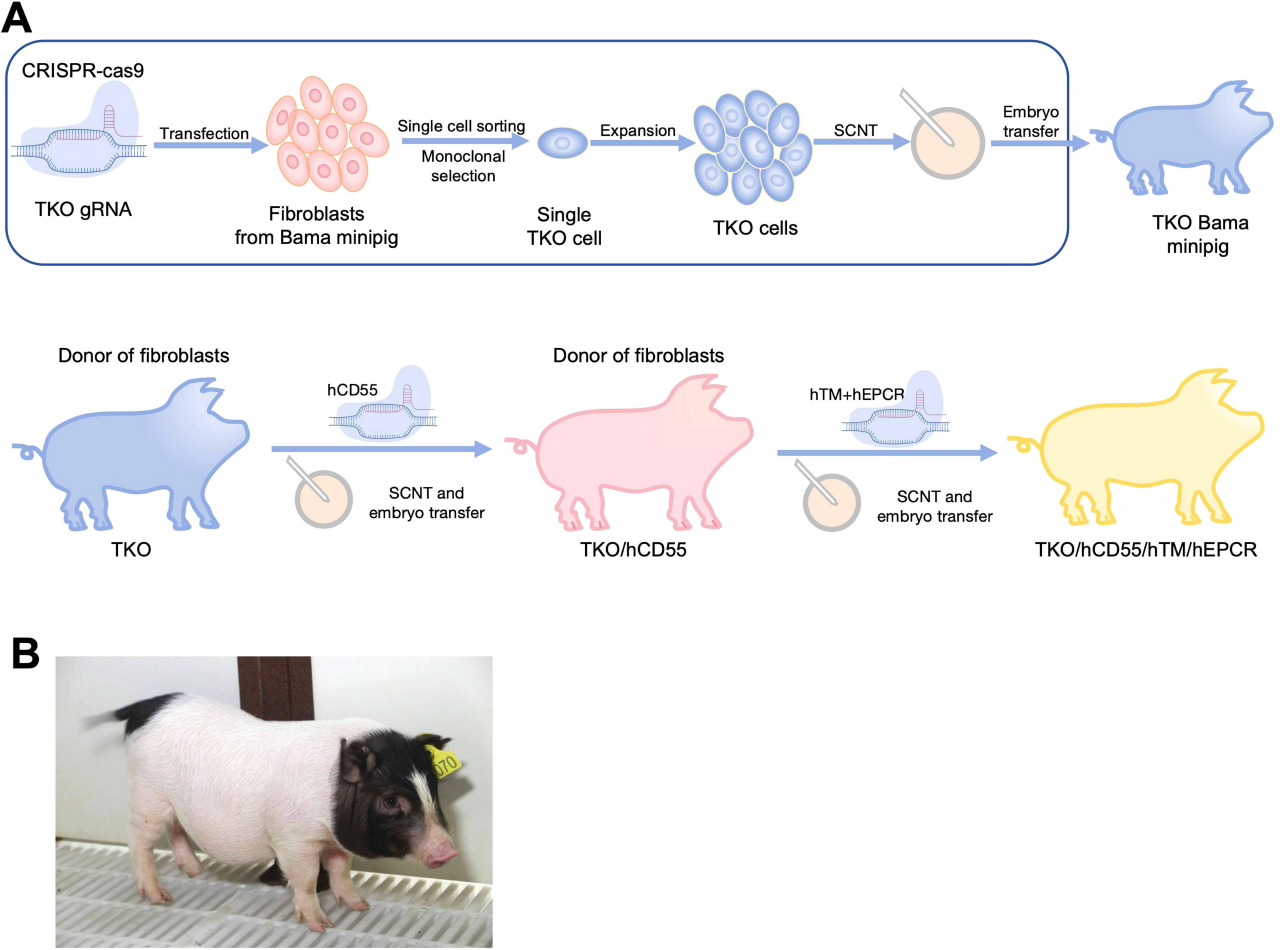
The generation of TKO/hCD55/hTM/hEPCR pigs. **(A)** The engineering workflow of TKO/hCD55/hTM/hEPCR pigs. WT Bama Ear fibroblasts, harvested from WT Bama minipigs, edited by Cas9 protein and gRNAs targeting GGTA1, CMAH and B4GALNT2. After single-cell sorting and monoclonal selection, the single cell carrying knockout of GGTA1, CMAH and B4GALNT2(TKO) was expanded and cloned into TKO pigs through SCNT and embryo transfer. Then, TKO/hCD55 were cloned under the TKO ear fibroblasts in the same procedures as TKO pigs. The TKO/hCD55/hTM/hEPCR(6GE) pigs were cloned using ear fibroblasts of TKO/hCD55 which edited by Cas9 and gRNAs targeting TM and EPCR. **(B)** A photograph of 6GE piglets.

**Figure 2:**
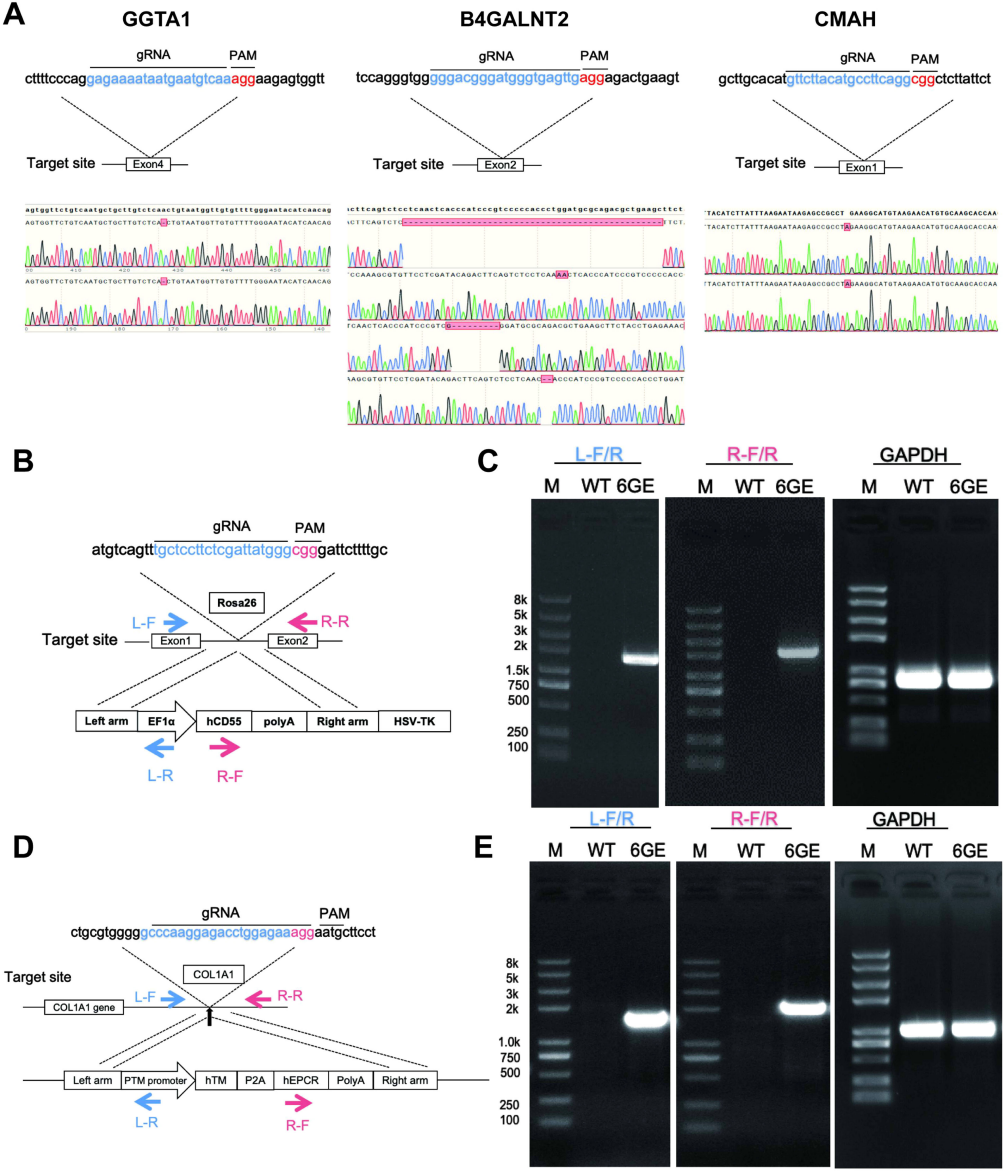
TKO/hCD55/hTM/hEPCR pig engineering and validation of the knockout genes and transgenes edits at the genomic level. **(A)** Knockout targets, gRNA sequences, and corresponding sequencing results for GGTA1, B4GALNT2, and CMAH. The knockout target for the GGTA1 gene is exon 4. Sequencing results indicate that the red box represents a -1, meaning a 1bp base deletion, resulting in a GGTA1 edit of (-1/-1). The knockout target for the B4GALNT2 gene is exon 2, with sequencing results as the first two chromatograms represent the sequencing results of the two chromosomes of B4GALNT2 (-49bp, +2bp), and the latter two chromatograms represent the sequencing results of the two chromosomes of B4GALNT2-like (-8bp, -2bp). The knockout target for the CMAH gene is exon 1, with the sequencing results of the two chromosomes of the CMAH gene being (+1bp/+1bp). **(B)** the transgenic strategy for CD55, involves the targeted insertion of the illustrated vector sequence into the Rosa26 locus. **(C)** the DNA identification results for CD55, with L-F/R and R-F/R serving as primers to identify the targeted integration of CD55 at the left and right ends of the integration site, respectively. **(D)** the transgenic strategy for TM-EPCR involves the targeted insertion of the vector sequence into the COL1A1 locus. **(E)**, the DNA identification results for TM-EPCR, with L-F/R and R-F/R serving as primers to identify the targeted integration of TM-EPCR at the left and right ends of the integration site, respectively, with GAPDH serving as the DNA internal control.

First, the engineering workflow of TKO/hCD55/hTM/hEPCR pigs was conducted and shown in **Figure 1A**. Three glycan epitopes (α-Gal, Neu5Gc and SDa) in pigs were knocked out using the CRISPR/Cas9 system (**Figure 2A**). We designed gRNAs for the fourth exon of the GGTA1 gene, the second exon of the B4GALNT2 gene, and the first exon of the CMAH gene. These vectors were used for transfection. GFP-positive cells were enriched by flow cytometry 48-72 hours after transfection, and DNA was extracted for amplification of the target genes to determine knockout efficiency. We identified gRNAs with high efficiency, and constructed gRNAs for all three genes onto a single knockout vector for transfection and selection of TKO cells. Subsequently, we selected two individual pigs, one male and one female, for cell line establishment and screening. Positive clones were obtained and used for SCNT and embryo transfer. Large white pigs were used as surrogate mothers for cloning, and TKO piglets were obtained. One week after birth, a small piece of ear tissue was collected from TKO piglets for DNA extraction to identify knockout genes. When the piglets were one month old, red blood cells, PBMCs, and endothelial cells were isolated to verify the expression level of α-Gal/SDa/Neu5Gc. The TKO pigs were used to establish ear fibroblast cell lines for the preparation of hCD55 transgenic pigs (**Figures 2B, C**). The hCD55 gene was inserted into the pig safe harbor Rosa26 locus using the CRISPR/Cas9 system and homologous recombination technology. Based on the sequence of the insertion site, homologous arms of about 1 kb were designed, located at both ends of the targeted insertion vector, and co-transfected with the Rosa26 knockout vector into TKO pig ear fibroblast cell lines. Positive single-cell clones were identified at the DNA level, and a single-cell positive clone was obtained for SCNT and embryo transfer, following the cloning method as described earlier. TKO/hCD55 piglets were obtained, and the insertion of CD55 was confirmed at the DNA level. After the birth of TKO/CD55 cloned pigs, they were raised until 6 months old and used for breeding to obtain F1 generation piglets. After the birth of F1 generation piglets, one female piglet was selected for ear fibroblast cell line establishment to establish hTM and hEPCR transgenic pigs (**Figure 2D, E**). The hTM and hEPCR transgenes were inserted into the pig genome safe harbor COL1A1 locus using the same integration method as hCD55. The expression of hTM and hEPCR was driven by the pig endogenous TM promoter. Combined with DNA-level identification, as shown in **Figure 1B**, we finally obtained TKO/hCD55/hTM/hEPCR piglets.

### 3.2 Assessment of α-Gal, SDa and Neu5Gc antigen and transgene hCD55, hTM and hEPCR expression

**Figure 3:**
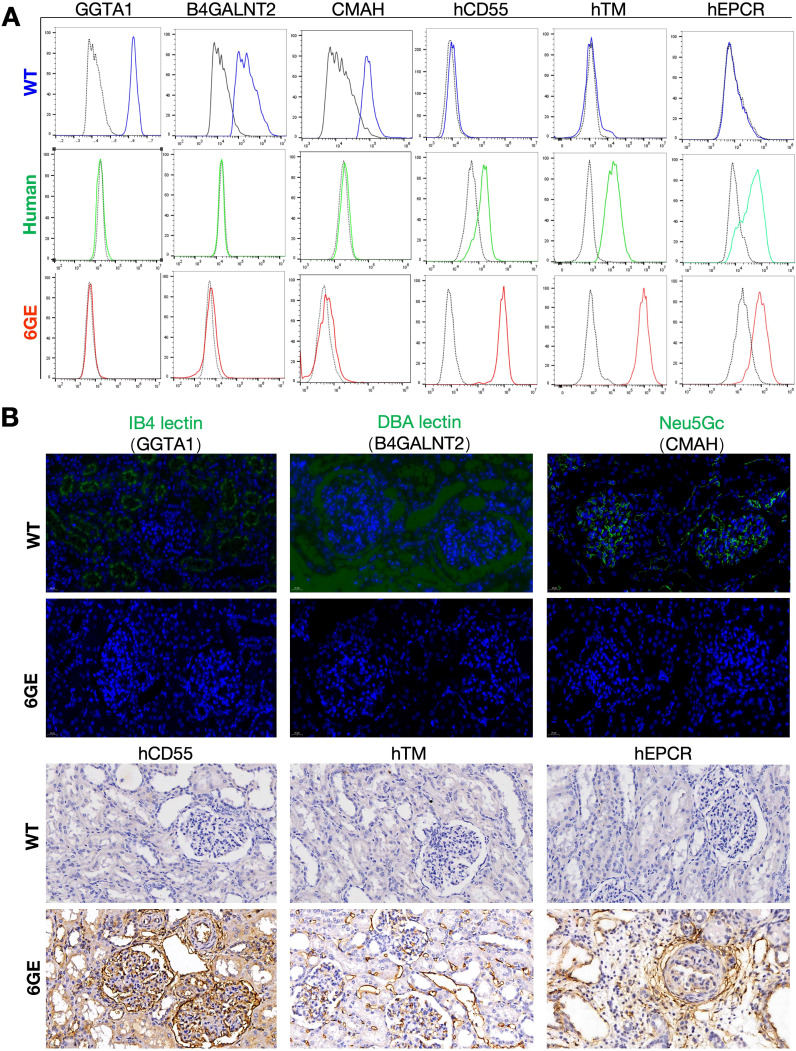
Validation of TKO/hCD55/hTM/hEPCR pigs at the protein levels. **(A)** FACS validation of GGTA1, B4GALNT2, CMAH, hCD55, hTM and hEPCR in 6GE pAECs, HUVECs and WT pigs were used as the control. **(B)** Immunofluorescence staining validation of GGTA1, B4GALNT2, CMAH, hCD55, hTM and hEPCR in kidney cryosections of 6GE pigs. Scale bars, 20 μm. Experiments were independently repeated three time.

After DNA sequencing and testing, the engineered genes in 6GE pigs were characterized on protein level. First, the WT pAECs and 6GE pAECs were harvested and cultured to confirm the deficiency of α-Gal, SDa and Neu5Gc antigens and expression of the transgenes hCD55/hTM/hEPCR using FACS analysis of HUVECs and pAECs from both cloned pigs and WT pigs. The α-Gal, SDa and Neu5Gc antigens were all negative in 6GE pigs when compared to that of WT pigs (**Figure 3A**), suggesting that the three genes for synthesizing these glycan epitopes in 6GE pigs were functionally eliminated. In contrast, hCD55/hTM/hEPCR showed significant higher expression level in 6GE pigs than in WT pigs. Especially, the expression level of hCD55 in 6GE pigs was almost 7 times higher than in humans (p<0.01), the hTM was 13 times higher than in humans (p<0.01), and the expression of hEPCR of 6GE pigs was similar to that in humans. We concluded that hCD55, hTM and hEPCR were all expressed successfully in 6GE pigs. The protein level of genetic modifications was further confirmed in kidney tissue using immunofluorescence staining for the three epitopes and Immunohistochemistry for transgenes hCD55/hTM/hEPCR. The results showed that expression of α-Gal, SDa and Neu5Gc antigens was totally negative in 6GE kidney tissue (**Figure 3B**) while these three epitopes were highly expressed in WT pigs. In contrast, the constitutive protein expression of transgenes hCD55/hTM/hEPCR was significantly detected in kidney tissue of 6GE pigs but not in WT pigs. Collectively, we concluded that our 6GE pigs were α-Gal, SDa and Neu5Gc antigen-knockout and had hCD55/hTM/hEPCR genetic modifications at the cellular and tissue level.

### 3.3 Human IgM and IgG antibody binding assay and cytotoxicity assay

**Figure 4:**
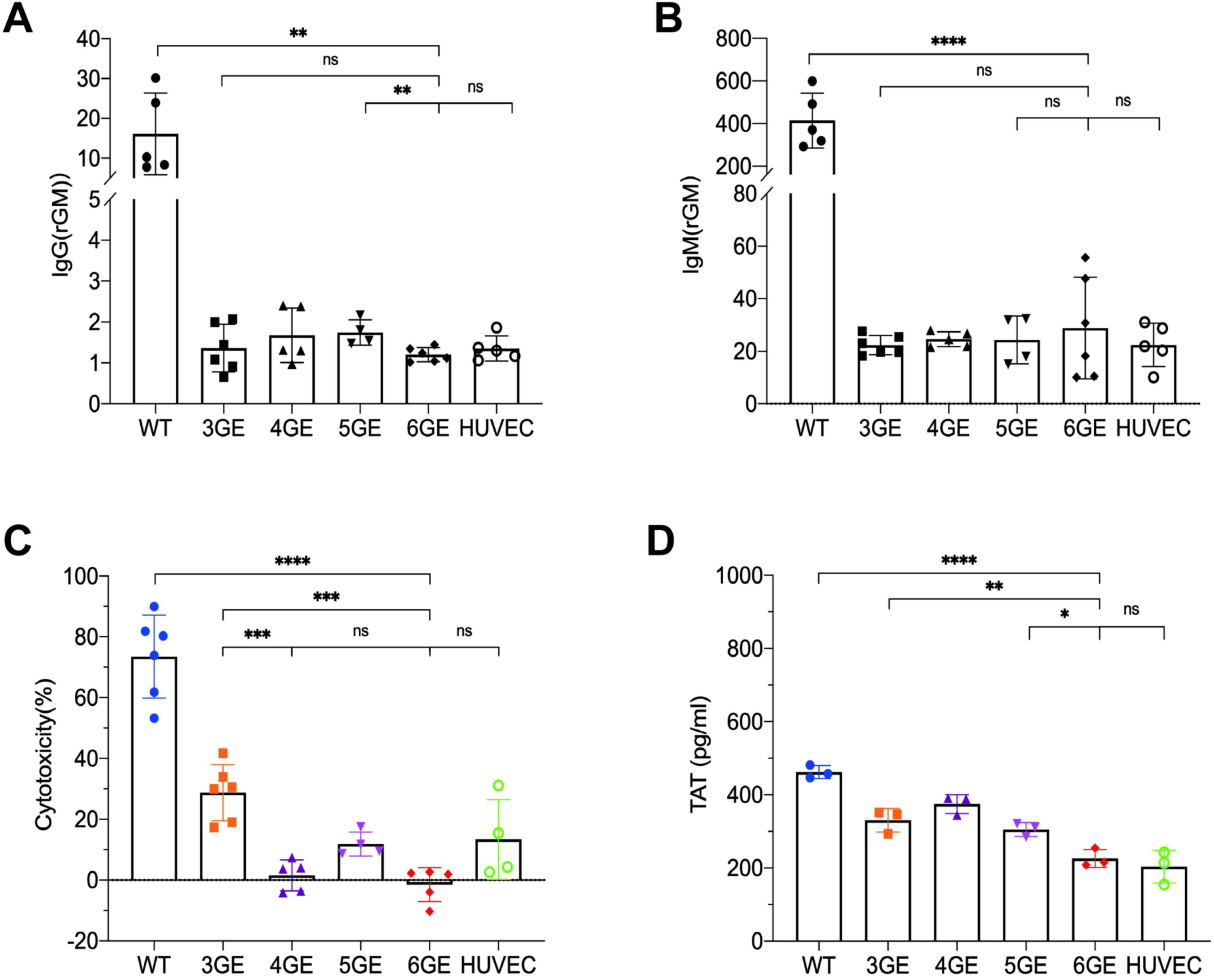
Functional validation of TKO/hCD55/hTM/hEPCR pigs in human antibody binding, complement toxicity and coagulation function. **(A)** The IgG expression of pAECs from WT, 3GE, 4GE, 5GE, 6GE binding to human serum, respectively. **(B)** Comparison of IgM expression of WT, 3GE, 4GE, 5GE and 6GE pAECs binding to human serum. HUVECs were used as the control. **(C)** Make a comparison of pAECs from WT, 3GE, 4GE, 5GE and 6GE antibody-dependent complement cytotoxicity to HUVECs of human. E, The TAT level expressed WT, 3GE, 4GE, 5GE, 6GE pAECs and HUVECs after incubation with whole human blood for an hour. Every point stand one sample and repeated for three times. rGM, relative IgM. ****P< 0.0001, ***P< 0.001, **P< 0.01, *P< 0.05; ns, not significant (P> 0.05).

To evaluate whether these gene modifications would reduce human serum immunoreactivity or not, pAECs were harvest from WT, 3GE, 4GE, 5GE, 6GE pigs, and HUVECs used as the control. The pAECs and HUVECs were incubated with a 25% concentration of heat-inactivated human serum, and the mean fluorescence intensity of IgM and IgG was determined. The 6GE group showed a significantly lower level of IgM and IgG binding than that of WT groups (IgG p<0.01, IgM p<0.0001) (**Figure 4A**), and there was no difference among 3GE pAECs, 6GE pAECs and HUVECs for the level of IgM and IgG. This indicated that knockout GGTA1, B4GALNT2, and CMAH minimized IgM and IgG binding of human serum. 6GE pigs showed significantly reduced immunoreactivity compared to WT pigs. Moreover, the cytotoxicity assay was used to confirm the potential benefit of hCD55 in donor organs. 4GE pAECs demonstrated significantly lower human complement cytotoxicity than 3GE pAECs (p<0.001) when incubated with a uniform pool of human serum complement. This was attributed to the high expression level of hCD55 in 4GE pigs (**Figure 4B**). Meanwhile, the cytotoxicity level of 6GE pigs showed no difference from the 4GE pigs (**Figure 4C**). Indeed, compared with controls, the porcine fibroblasts expressing hCD55 significantly reduced complement-dependent cytotoxicity.

### 3.4 Coagulation system

TM or EPCR were demonstrated to be associated with reduced platelet activation/aggregation and to induce a state of anticoagulation. To test the function of the transgenes of hTM and hEPCR in 6GE pigs, the TAT assay was used. The pAECs from WT, 3GE, 4GE, 5GE, 6GE pigs were seeded and coculture with fresh human whole blood, the TAT value of supernatant was tested. The result is shown in **Figure 4D**. 6GE reduces the formation of TAT complexes to significantly lower levels than of WT or TKO (p<0.0001, p<0.01, respectively). We also found that 6GE pAECs significantly reduced the TAT level more than 5GE pAECs (p<0.05). The results suggested that 6GE pigs acquired enhanced coagulation compatibility with human factors, and that hTM and hEPCR should be inserted in combination to get a better anticoagulation result than hTM alone.

### 3.5 Pathophysiology condition

**Figure 5:**
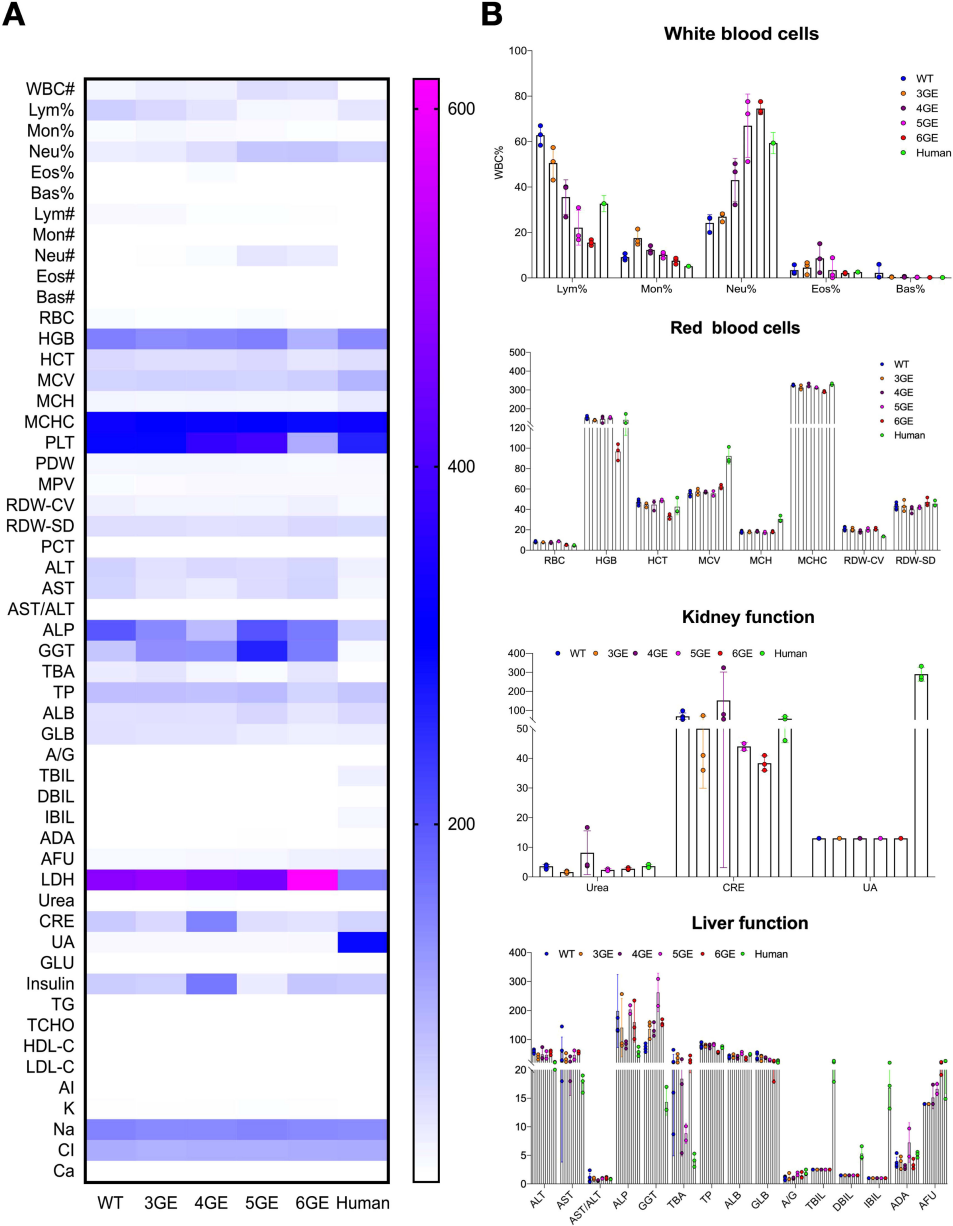
The pathophysiology of TKO/hCD55/hTM/hEPCR pigs. **(A)** Comparison of the pathophysiology of WT, 3GE, 4GE, 5GE and 6GE pigs and humans. **(B)** The results of white blood cells, red blood cells, liver function and kidney function among WT, 3GE, 4GE, 5GE and 6GE pigs. Every point stand for one sample.

To assess the pathophysiology of 6GE pigs, the blood of WT pigs, 6GE pigs and humans were collected. The overall comparisons between WT, 3GE, 4GE, 5GE, 6GE pigs and human were shown in **Figure 5A**. After that, the white blood cells, red blood cells, liver function and kidney function were compared (**Figure 5B**). For white blood cells, the results showed that the proportion of neutrophils in each individual in the order of small to large: 6GE, 5GE, 4GE, 3GE, and WT. The 6GE pigs exhibited the highest percentage of neutrophils and demonstrated increased susceptibility to infections under general environmental conditions. To investigate the types of infection present in 6GE pigs, we collected oral and nasal swab samples for analysis. The results identified bacterial infections, including *Streptococcus suis*, which were primarily associated with the respiratory system and did not involve in porcine aortic endothelial cells. After summarizing, the ease of infection from easy to difficult was 6GE, 5GE, 4GE, 3GE, and WT. Additionally, The LDH level in 6GE pigs was significantly higher than WT but no different with TKO/CD55/TM. According to the proportion of neutrophils data in **Figure 5B**, the higher LDH level may be ascribed to the bacterial infection. However, LDH levels in 6GE is still in the normal range. therefore, even if the LDH of 6GE is slightly increased, it will not have much impact on the physiology of 6GE pigs. For the red blood cells, the MCV and MCH of gene-edited pigs were lower than of humans, but the counts of red blood cells in gene-edited pigs were higher than in humans. The Hgb and platelet counts of 6GE pigs showed significantly lower levels than in humans, suggesting that the insertion of hEPCR is a key factor affecting coagulation function of 6GE pigs. According to blood tests, we also observed normal vital organ functions in the liver and kidneys of our 6GE pigs, and these organs fully met human needs.

## 4 Discussion

Based on current immunological analyses of pig-to-monkey and clinical xenotransplantation outcomes, endothelial activation, complement activation, and coagulation system disorders are identified as significant factors contributing to rejection. Therefore, the most basic combination of donor pig genes includes triple antigen knockout, expression of hCRPs and anti-coagulation-related genes. These gene modifications are ideal candidates for human transplantation strategies (28, 29). We obtained TKO/hCD55/hTM/hEPCR pigs using genome editing and F1 generation breeding. Through *in vitro* experiments with human serum, such as IgM and IgG binding assays and human complement cytolysis, we demonstrated that these 6GE pigs are ideal donor pigs for organ or tissue-specific xenotransplantation. 6GE pigs are promising donors for clinical xenotransplantation.

Multiple gene editing is associated with some side effects such as abnormal physiological states, slow organ growth, difficulties in breeding, etc. The 6GE pigs mentioned in this study were obtained through a combination of gene-editing and breeding. Using this approach, genetically edited pigs are characterized by greater genetic stability, safety, and effectiveness. The gene expression in subsequent generations of multi-gene-edited pigs were tested and showed that our inheritance patterns and phenotype were stable from generation to generation. For example, in TKO/CD46/CD55/TM gene-edited pigs, the expression level of CD55 had no big difference among Filial generation(F)0, F1 and F2. Additionally, the location, gene copy number, inheritability, and expression level of these transgenes can be clearly elucidated. We primarily selected superior F1 generation individuals of gene-edited pigs based on observations and evaluations of each round of genome editing and pig production, considering factors such as the pigs’ appearance, growth rate, physiological status, and genetic stability. This method ensures the acquisition of genetically and phenotypically stable and healthy gene-edited pigs. Therefore, all edited genes of the TKO/hCD55/hTM/hEPCR pigs in our study have been fully confirmed and interpreted, confirming complete knockout of antigen genes and stable, high expression levels of the transferred genes.

Currently, the gene-edited pigs used in clinical transplantation were typically involve multiple edited genes. The NYU team completed heart transplants from ten-gene edited pigs into brain-dead donors, ischemic damage to myocardial cells occurred in one of cases due to the relatively small size of the pig heart. Although they utilized growth hormone receptor-knockout (GHRKO) pigs, the stability and consistency of controlling pig organ size by knocking out the growth hormone receptor gene remains unclear (30). Therefore, selecting minipigs as the donor pigs, the organ sizes closer to those of humans, preventing to knock out GHR might be a better option. Additionally, in a case of xenotransplantation completed by the UMSOM research team, porcine cytomegalovirus (PCMV) was detected, indicating the importance and necessity of removing PCMV to prevent destructive inflammatory reactions (31). eGenesis has also directly knocked out genes in pigs to achieve PERVKO (32), while the latest MGH study on kidney transplantation from genetically modified pigs to humans involved editing over 50 genes to silence inactivating porcine endogenous retroviruses (26). The 6GE pigs used in the present study were Bama minipigs, characterized by organs similar in size to humans and lacking the PERV-C subtype, it is known that PERV in infected human cells is a recombinant PERV-A/C which is driven by PERV-C. Therefore, editing fewer genes can address cost concerns.

The expression levels of a gene will affect the result of an organ xenotransplant. The factors contributing to rejection in the first case of heart xenotransplantation completed by the UMSOM research team were extensive damage to endothelial cells and capillaries, leading to interstitial edema, erythrocyte extravasation, and complement deposition, suggesting antibody-mediated rejection. Although 10-gene pigs were used, the expression levels of CD55 and CD46 may not have been sufficiently high to achieve the desired outcomes (33). This discrepancy might be related to the expression levels of the transferred genes. In our 6GE pigs, the expression level of hCD55 in Peripheral blood is seven times higher than in humans, with *in vitro* results showing a CDC close to zero, suggesting that the higher expression levels of hCD55 can indeed reduce CDC. For coagulation system, the expression of hTM and hEPCR is also varied and controversial. High hTM expression showed significantly anti-aggregation effects and prolonged survival of solid organ xenografts (34). Although both hTM and hEPCR have anti-coagulation properties, some studies expected that co-expressed hTM and hEPCR (21) but some may do not (35). hEPCR enhances hTM’s cofactor activity and amplifies its cytoprotective effects (36). Recent studies with 69-gene pigs in kidney xenotransplantation showed that overexpression of TM and EPCR resulted in lower TAT levels compared to WT or TKO pigs (37). Our experiments found that 6GE pigs with higher hTM and similar hEPCR levels to humans had significantly reduced TAT levels compared to 5GE pigs. This indicates that co-expression of hEPCR and hTM is more effective at anti-aggregation than hTM alone, and excessive hEPCR does not needed. Therefore, the high level of transferred gene does not inherently guarantee improved outcomes. Further validation is required to elucidate the relationship between expression levels and the efficacy of transferred genes, particularly in the context of multiple gene edits.

While TKO/hCD55 pigs are considered safe when they were all bred in a general environment, the 6GE pigs are easily infected in general environments infection, the factors may be focusing on three key aspects: (i) gene editing, (ii) individual variability, and (iii) environmental conditions. i)Gene editing: The impact of gene editing on infection risk primarily depends on the specificity of the edited genes. Evidence from existing studies suggests that certain pathogens can exploit complement receptors to invade host cells. For instance, coxsackievirus and other enteroviruses utilize Decay Accelerating Factor (DAF or CD55) as a receptor(PMID: 8764022), introducing hCD55 into TKO pigs (TKO/hCD55 clone pig) could potentially elevate the risk of viral and bacterial infections. For hTM, no literature has yet reported their roles as targets for viral or bacterial pathogens. hEPCR regulated the response to acute infection and also as a marker in patients with acute infections as well as in patients with vascular diseases. (PMID 37846891). According to the data we summarized, TKO/hCD55 pigs previously maintained under conventional environmental conditions for multiple generations did not exhibit signs of infection. TKO/hCD55 with transgene hTM and hEPCR were get infected easily maybe ascribe the hTM/hEPCR or this multiple-genes combination, there is currently no evidence to explain this issue. ii) Individual variability: Infection susceptibility may also vary due to individual differences, potentially linked to the genetic background of the F1 generation. To generate the gene-edited pigs, the cell line establishment and screening when the F1 pig was one week of age. However, whether changes in genetic background could lead to infection susceptibility later in life remains uncertain. Notably, we have developed other multi-gene-edited pigs, such as TKO/CD55/CD46/TM pigs, which have shown no evidence of infection. This observation recently was published (PMID: 39317190) and explained that pre-experimental testing confirming the absence of viral or bacterial infections in TKO/CD55/CD46/TM pigs. iii) Environmental conditions: The living environment plays a critical role in maintaining pig health. As everyone knows, if the viruses or bacteria in the environment exceed standards, these gene-edited pigs will be more susceptible to infection or illness. Therefore, environmental conditions are also one of the very important influencing factors. As highlighted earlier, TKO/CD55 and TKO/CD55/CD46/TM pigs raised under the same conditions showed no signs of infection, suggesting that general housing environments are generally safe. However, some gene locus or multiple gene edited would be the potential targets for viral or bacterial pathogens, so this part need further and more investigation to make sure that the healthy donors from 6GE pigs would be safe for the clinical use.

There are several limitations in the present study. First, further *in vivo* studies are necessary to test the efficacy of 6GE pig grafts in NHPs to validate the overall genetic combination and its functional efficacy. Furthermore, based on current clinical research findings, besides necessary gene editing, the impact of immunosuppressive drugs on graft survival is crucial. Therefore, the development of appropriate immunosuppressive regimens is essential to address issues such as inflammation and coagulation observed in xenotransplantation. Trials of pig-to-NHP transplantation have demonstrated that blocking the CD40/CD154 pathway significantly prolongs survival (38, 39). Additionally, complement inhibition is vital for suppressing early rejection in xenotransplantation. Anti-C5 (e.g., tesidolumab) can effectively reduce the formation of membrane attack complexes induced by early IgM antibodies (40). As xenotransplantation preclinical studies progress, the risk of cross-species microbial infection, particularly porcine cytomegalovirus (PCMV), has received increasing attention. Several studies have shown that PCMV transmission correlates with recipient survival time, and PCMV activation may exacerbate immune rejection. ALL the above suggesting that (i) the more gene editing carried out, the greater the health risk to the pig and reproduction becomes more difficult; (ii) since polygenic pigs are susceptible to infection in ordinary environments, pigs for clinical application should be bred in designated pathogen-free (DPF) facilities. Therefore, the breeding of ‘clean’ pigs is a prerequisite for conducting preclinical research.

Despite these limitations, pigs obtained through gene editing and breeding offer advantages such as genetic stability and clarified gene functions. *In vitro* experiments have demonstrated that high expression of hCD55, along with the co-expression of the hEPCR and hTM genes, is expected to effectively reduce the human complement cytotoxicity and enhance anticoagulant efficacy in genetically modified pigs. TKO/hCD55/hTM/hEPCR pigs achieve maximum compatibility with humans but with minimal gene combinations, while considering biosafety, it is very necessary to recommend that multi-gene edited pigs be raised in DPF facilities. So that they would be ideal donors for preclinical and clinical studies of xenotransplantation, facilitating the accumulation of technical and foundational research experience to accelerate clinical application.

## Data Availability

The original contributions presented in the study are included in the article/supplementary material. Further inquiries can be directed to the corresponding author.

## Glossary

GGTA1: Glycoprotein alpha-galactosyltransferase 1
CMAH: Cytidine monophospho-N-acetylneuraminic acid hydroxylase
B4GALNT2: Beta-1,4-N-acetyl-galactosaminyltransferase 2
hCD55: Decay accelerating factor (DAF)
CD46: membrane cofactor protein
CD59: MAC-inhibitory protein
TM: Thrombomodulin
EPCR: Endothelial protein C receptor
TKO: α-Gal, Neu5Gc and SDa KO
3GE: TKO
4GE: TKO/hCD55
5GE: TKO/hCD55/hTM
6GE: TKO/hCD55/hTM/hEPCR
pAEC: pig Arterial Endothelial Cells
TAT: Thrombin-Antithrombin Complex
HAR: Hyperacute Rejection
hCRPs: human complement regulatory proteins
SCNT: Somatic cell nuclear transfer
OWM: Old World monkey
HUVEC: Human Umbilical Vein Endothelial C
WBC: White Blood Cell
Lym: Lymphocyte
Mon: Monocyte
Neu: Neutrophil
Eos: Eosinophil
Bas: Basophil
RBC: Red Blood Cell
HGB: Hemoglobin
HCT: Hematocrit
MCV: Mean Corpuscular Volume
MCH: Mean Corpuscular Hemoglobin
MCHC: Mean Corpuscular Hemoglobin Concentration
PLT: Platelet
PDW: Platelet Distribution Width
MPV: Mean Platelet Volume
PCT: Procalcitonin
BUN: Blood Urea Nitrogen
CRE: Creatinine
UA: Uric Acid
ALT: Alanine Aminotransferase
AST: Aspartate Aminotransferase
ALP: Alkaline Phosphatase
GGT: Gamma-Glutamyl Transferase
TBA: Total Bile Acid
TP: Total Protein
ALB: Albumin
GLB: Globulin
TBIL: Total Bilirubin
IBIL: Indirect Bilirubin
ADA: Adenosine Deaminase
AFU: Alpha-L-Fucosidase
LDH: Lactate Dehydrogenase.
